# A nationwide survey of clinical characteristics, management, and outcomes of acute kidney injury (AKI) – patients with and without preexisting chronic kidney disease have different prognoses

**DOI:** 10.1097/MD.0000000000004987

**Published:** 2016-09-30

**Authors:** Heng-Chih Pan, Pei-Chen Wu, Vin-Cent Wu, Ya-Fei Yang, Tao-Min Huang, Chih-Chung Shiao, Te-Chuan Chen, Der-Cherng Tarng, Jui-Hsiang Lin, Wei-Shun Yang, Chiao-Yin Sun, Chan-Yu Lin, Tzong-Shinn Chu, Mai-Szu Wu, Kwan-Dun Wu, Yung-Chang Chen, Chiu-Ching Huang

**Affiliations:** a Division of Nephrology, Department of Internal Medicine, Keelung Chang Gung Memorial Hospital, Keelung; b Division of Nephrology, Department of Internal Medicine, Mackay Memorial Hospital; c Division of Nephrology, Department of Internal Medicine, National Taiwan University Hospital, Taipei; d Division of Nephrology, Department of Internal Medicine, China Medical University Hospital, Taichung; e Division of Nephrology, Department of Internal Medicine, National Taiwan University Hospital Yun-Lin Branch, Yunlin; f Division of Nephrology, Department of Internal Medicine, Saint Mary's Hospital Luodong; and Saint Mary's Medicine, Nursing and Management College, Yilan; g Division of Nephrology, Department of Internal Medicine, Kaohsiung Chang Gung Memorial Hospital, Kaohsiung; and Chang Gung University College of Medicine, Taoyuan; h Division of Nephrology, Department of Internal Medicine, Taipei Veterans General Hospital, Taipei; i Division of Nephrology, Department of Internal Medicine, Taoyuan General Hospital, Ministry of Health and Welfare, Taoyuan; j Division of Nephrology, Department of Internal Medicine, National Taiwan University Hospital Hisn-Chu Branch, Hsin-Chu City; k Kidney Research Center, Department of Nephrology, Chang Gung Memorial Hospital; and Chang Gung University College of Medicine, Taoyuan; l Division of Nephrology, Department of Internal Medicine, Taipei Medical University Hospital, Taipei, Taiwan (R.O.C.).

**Keywords:** AKI, CKD, prognosis, SCr

## Abstract

Acute kidney injury (AKI) is a common complication in hospitalized patients. The International Society of Nephrology implemented the “0 by 25” initiative aimed at preventing deaths from treatable AKI worldwide by 2025 and conducted a global snapshot survey in 2014. We joined in the project and conducted this study to compare the epidemiology, risk factors, and prognosis between patients with pure AKI and those with acute-on-chronic kidney disease (ACKD). In this study, we prospectively collected demographic parameters and data on clinical characteristics, baseline comorbidities, management, and outcomes of 201 AKI patients in 18 hospitals in Taiwan from September 2014 to November 2014. The in-hospital mortality rate was 16%. AKI was mostly attributed to sepsis (52%). Multivariate logistic regression indicated that oliguria was a positive independent predictor of in-hospital mortality, whereas preexisting CKD and exposure to nephrotoxic agents were negative independent predictors. The prevalence of vasopressor use, intensive care unit care, and mortality were significantly higher in pure AKI patients than in ACKD patients. Moreover, serum creatinine (SCr) levels significantly increased within 7 days after AKI diagnosis in nonsurvivors but not in survivors in the pure AKI group. By contrast, SCr levels were persistently lower in nonsurvivors than in survivors in the ACKD group during the same period. We thus determined that the prognosis of ACKD patients differed from that of pure AKI patients. Considering the CKD history in the future AKI staging system may improve prognosis prediction.

## Abstract 2

Supplemental Digital Content is available in the text

## 1 Introduction

The incidence rate of acute kidney injury (AKI) among hospitalized patients is increasing.^[1]^ Pathophysiological factors associated with AKI are also implicated in the dysfunction of other organs, indicating that AKI is often part of multiple organ failure syndrome.^[2,3]^ In the literature, AKI occurrence is associated with higher risks of multiple comorbidities and chronic kidney disease (CKD) as well as higher short-term and long-term mortality.^[4–7]^ In addition to the associated health impact, AKI increases in-hospital and posthospitalization resource utilization.^[8,9]^ The collaborative campaign World Kidney Day 2013 promoted by the International Society of Nephrology and International Federation of Kidney Foundations highlighted that AKI is presently a major global health concern.^[10,11]^

To standardize the definition of AKI and facilitate advances in clinical practise and research, the Kidney Disease: Improving Global Outcomes (KDIGO) group reconciled the definition of Risk, Injury, Failure, Loss of Kidney function, and End-stage Kidney Disease and the Acute Kidney Injury Network classification systems. To date, several studies have adequately validated the prediction sensitivity and accuracy of the KDIGO classification system.^[12–15]^ However, the specificity of the serum creatinine (SCr)-based AKI definition is uncertain, particularly when applied to patients with preexisting CKD.^[16]^

Recently, the International Society of Nephrology implemented the “0 by 25” initiative and conducted a global snapshot project to survey the incidence, risk factors, etiologies, diagnosis, and outcomes of AKI worldwide during a 6-week period in 2014. The Consortium for Acute Kidney Injury and Renal Diseases, representing a party of nephrologists and intensivists from 30 medical centres in Taiwan,^[17]^ participated in the project and conducted this original nationwide study to test the hypothesis that clinical characteristics and prognosis are different between AKI patients with and without preexisting CKD under the current AKI definition of KDIGO classification system. The secondary objective of this study was to explore the risk factors, contributors, resource utilization, and outcomes of AKI in Taiwan.

## 2 Materials and methods

### 2.1 Participants

This study included 201 AKI patients aged between 20 and 85 years in 18 hospitals in Taiwan from September 2014 to November 2014. Patients who received chronic dialysis were excluded.

### 2.2 Data collection

Baseline creatinine was defined as follows: the lowest SCr level in outpatient department (OPD) settings within 3 months before admission, the latest creatinine level during OPD follow-up for patients who did not visit an OPD within 3 months before admission, and the lowest creatinine level before dialysis during the index admission for those whose previous SCr levels were unknown. Baseline albumin and baseline total cholesterol were defined as follows: the lowest serum albumin and total cholesterol levels in OPD settings within 3 months before admission and the latest albumin and total cholesterol levels during OPD follow-up for patients who did not visit an OPD within 3 months before admission. AKI was diagnosed according to the KDIGO-AKI guideline,^[18]^ and CKD was diagnosed based on history, baseline SCr (>1.5 mg/dL), proteinuria (>300 mg/day), and abnormal renal ultrasound findings (such as abnormal renal echogenicity, kidney size, and cortical thickness) before hospitalization.^[19]^ Pure AKI was defined as AKI that developed in patients without preexisting CKD, and acute-on-chronic kidney disease (ACKD) was defined as AKI that developed in those with preexisting CKD. After identifying AKI, we enrolled patients in this study. All personal information was encrypted in the database, and there was no breach of privacy or interference with clinical decisions. Hence, the local institutional review board of each hospital and National Research Program for Biopharmaceuticals approved this protocol and waived the need for informed consent (Approval No. NRPB2014050014).

We prospectively collected demographic data and data on risk factors for AKI, which were associated symptoms and signs, SCr and BUN levels, urine amount, contributors of AKI, indication of renal replacement therapy, renal replacement therapy modality, and hospital survival and renal outcomes. SCr and BUN levels and the urine amount were collected at 5 time points (at onset of AKI, peak of AKI, before dialysis initiation, 7th day of AKI, and at hospital discharge).

### 2.3 Statistical analyses

Continuous variables were summarized as means and standard derivations unless otherwise stated. In the primary analysis, we compared hospital survivors with nonsurvivors. All variables were tested for normal distribution by using the Kolmogorov–Smirnov test. Student *t* test was used to compare the means of continuous variables and normally distributed data; otherwise, the Mann–Whitney *U* test was used. The χ^2^ test was used to compare the categorical data. The risk factors were assessed through univariate analysis by using the Cox proportional hazard model, and statistically significant (*P* < 0.05) variables identified through univariate analysis were included in multivariate analysis by applying multiple logistic backward regression analysis to obtain variables independently correlated with in-hospital survival. The SCr levels, measured at baseline, onset of AKI, peak of AKI, and 7th day of AKI, were compared between in-hospital survivors and nonsurvivors by using repeated-measures analysis of variance with the general linear model procedure. All statistical tests were 2-tailed, and a value of *P* < 0.05 was considered statistically significant. Data were analyzed using Statistical Package for the Social Sciences software, version 19.0, for Windows (SPSS Inc., Chicago, IL).

## 3 Results

### 3.1 Patient characteristics

We enrolled 201 AKI patients in this study. The overall in-hospital mortality rate was 15.9% (32/201). Table 1 shows demographic data and clinical characteristics of the patients. The mean patient age was 68 years, and the mean length of hospital stay was 16 days. Among the 201 patients, 65.2% were male, and 35.8% had preexisting CKD. The in-hospital mortality rates of AKI patients with preexisting CKD (acute-on-chronic disease, ACKD group) and without CKD (pure AKI group) were 5.6% (4/72) and 21.7% (28/129), respectively. The ACKD group comprised older patients who were more frequently managed by nephrologists and had a higher percentage of diabetes mellitus, whereas the pure AKI group involved patients with a higher percentage of chronic liver disease.

**Table 1:**
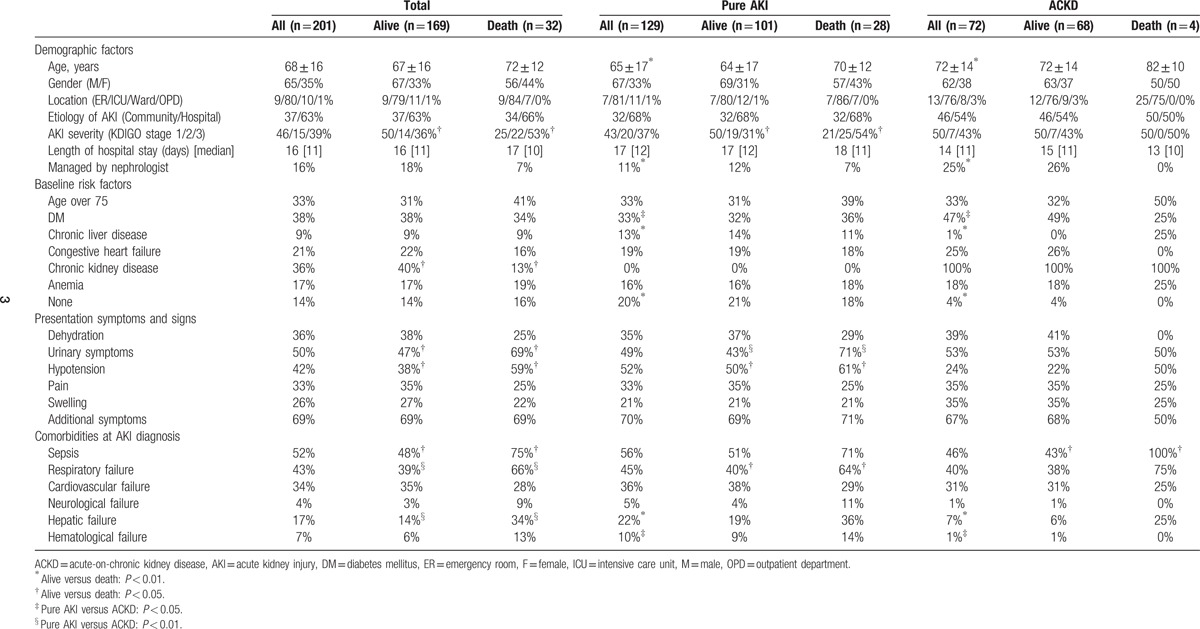
Clinical characteristics according to in-hospital mortality.

Comparing the survivors and nonsurvivors of total 201 patients, there was no significant difference in age or gender. The nonsurvivors were more likely to have urinary symptoms, hypotension, sepsis, respiratory failure, and hepatic failure at AKI diagnosis, while the survivors were more likely to have past history of chronic kidney disease. In the pure AKI group, the nonsurvivors had a higher percentage of urinary symptoms, hypotension, and respiratory failure than the survivors, while in the ACKD group, the nonsurvivors had a higher percentage of sepsis than the survivors.

Table 2 presents the renal characteristics of the patients. Sepsis was the major contributor of AKI (52%). Compared with nonsurvivors, survivors in all patients and ACKD group exhibited significantly higher SCr levels at AKI diagnosis. The prevalence of hepatic disease, sepsis, systemic diseases, and nonrenal neoplasm-related AKI was higher in nonsurvivors, and the prevalence of AKI attributable to nephrotoxic agents was significantly higher in survivors. In addition, the prevalence of oliguria and fluid overload was higher in nonsurvivors than in survivors. This trend was also observed in the ACKD group but not in the pure AKI group. Notably, severity of AKI was independently associated with in-hospital mortality.

**Table 2:**
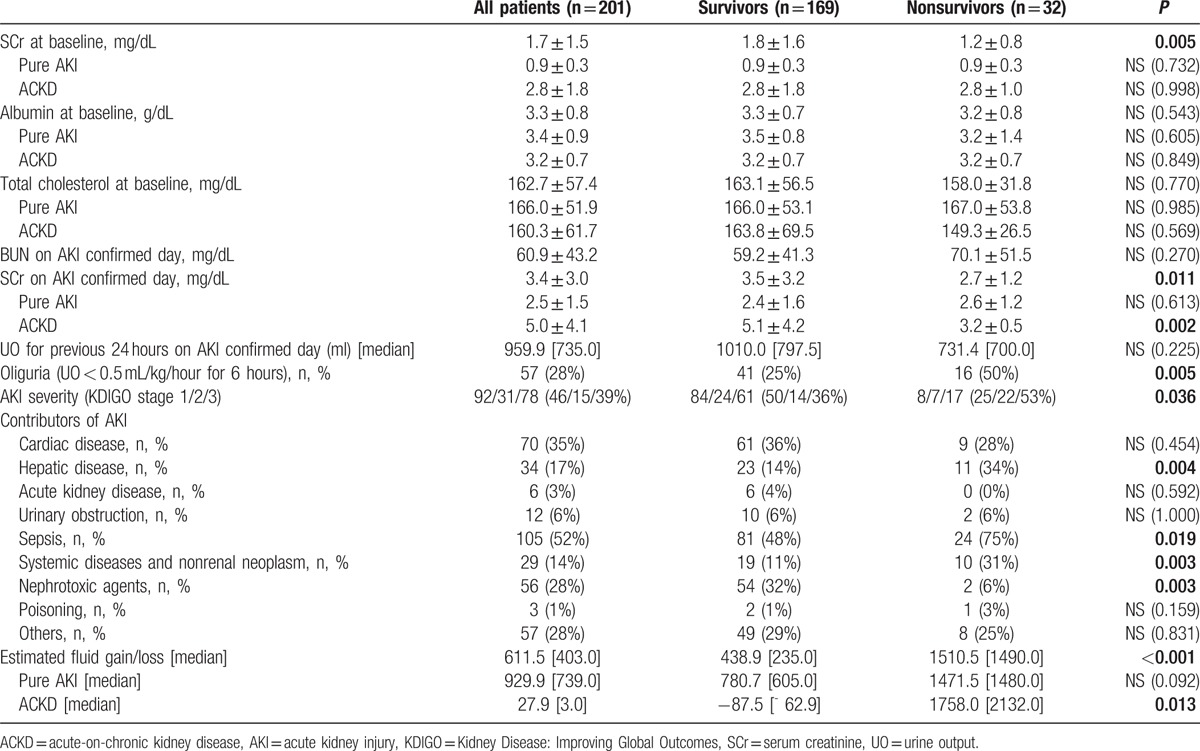
Kidney function-related clinical parameters of survivors and nonsurvivors.

At AKI diagnosis, most patients required fluid replacement therapy. The use of albumin was significantly higher in nonsurvivors. Forty percent of patients required diuretics and 31% required dialysis. The use of vasopressors and urinary diversions was significantly higher in nonsurvivors. Comparing the indication for dialysis revealed that severe electrolyte or acid–base disturbances were significantly higher in nonsurvivors. Type and duration of dialysis were not significantly different between the survivors and nonsurvivors. The SCr level before dialysis initiation was significantly higher in survivors than in nonsurvivors among the all patients and ACKD group. The deterioration and recovery of renal function within 7 days after AKI diagnosis were significantly associated with mortality. The nonsurvivors were more likely to have deterioration or stable of renal function while the survivors were more likely to have complete or partial recovery of renal function (Table 3).

**Table 3:**
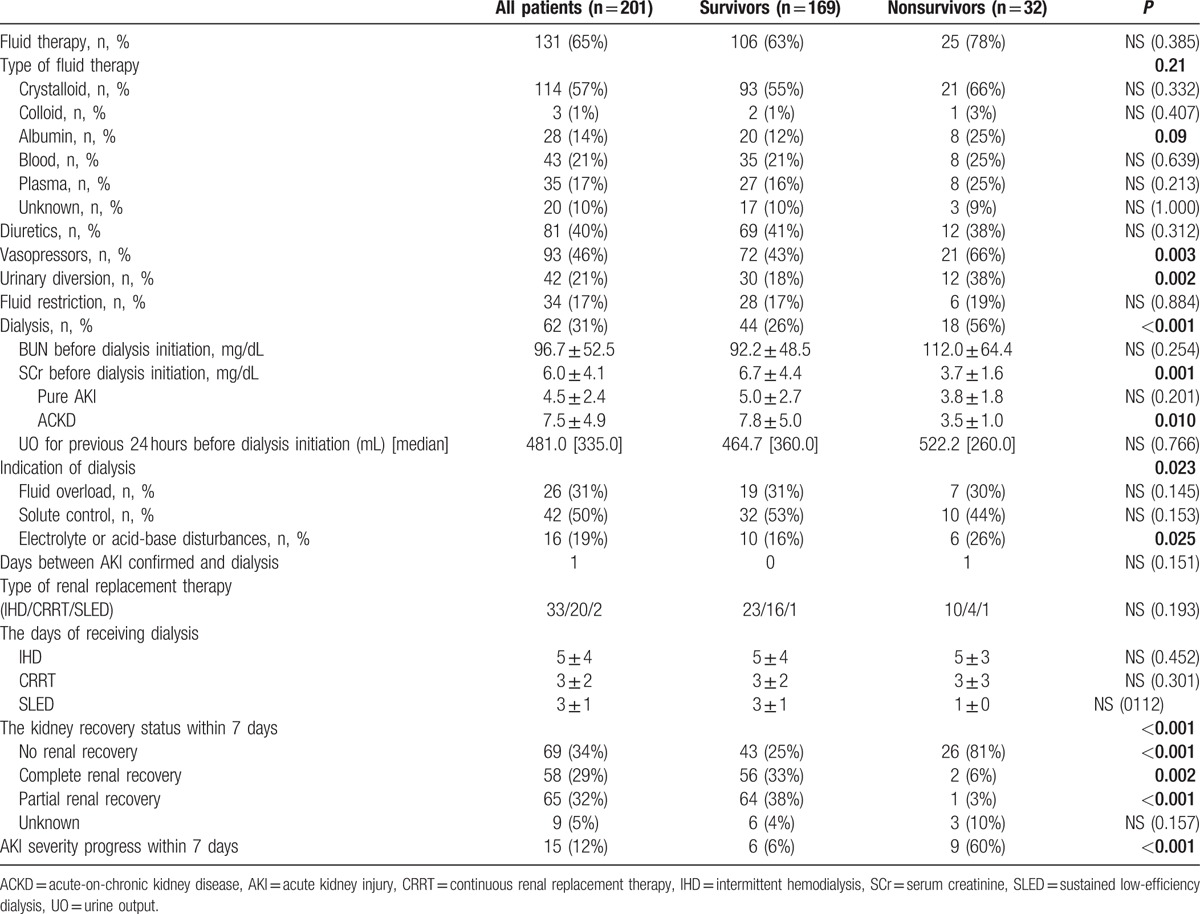
Management and outcomes according to in-hospital mortality.

### 3.2 Risk factors for in-hospital mortality

Univariate analysis showed that 10 (Table 4) of the 42 variables (Tables 1 and 2) were good prognostic indicators. Multivariate analysis revealed that the preexisting CKD, AKI attributable to nephrotoxic agents, and oliguria occurrence at AKI diagnosis were independent significant prognostic indicators of in-hospital mortality (Table 4). The preexisting CKD and AKI attributable to nephrotoxic agents were associated with a lower probability of mortality, whereas oliguria occurrence at AKI diagnosis was associated with a higher probability of mortality.

**Table 4:**
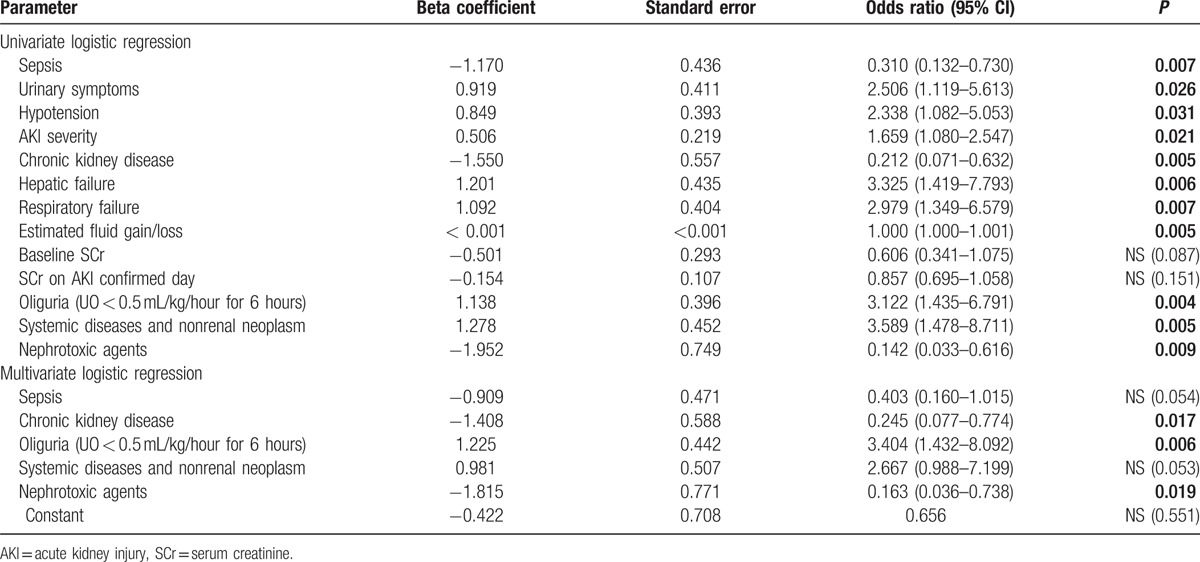
Variables at AKI diagnosis showing prognostic significance for in-hospital mortality.

### 3.3 Management and outcomes

Among the 3 AKI severities, patients with KDIGO stage 1 demonstrated a significantly lower probability of vasopressor use and death and a higher probability of being transferred out from the intensive care unit. These trends were also noted in the pure AKI group but not in the ACKD group. Patients with KDIGO stage 3 were significantly associated with a higher probability of receiving dialysis, becoming dialysis dependent, dying from cardiovascular events, and exhibiting higher SCr levels at hospital discharge. In the ACKD group, the patients with KDIGO stage 2 and 3 had significantly higher scheduled follow-up rates within 3 months after discharge. However, the scheduled follow-up rates of the 3 AKI severities were similarly low in the pure AKI group (Table 5).

**Table 5:**
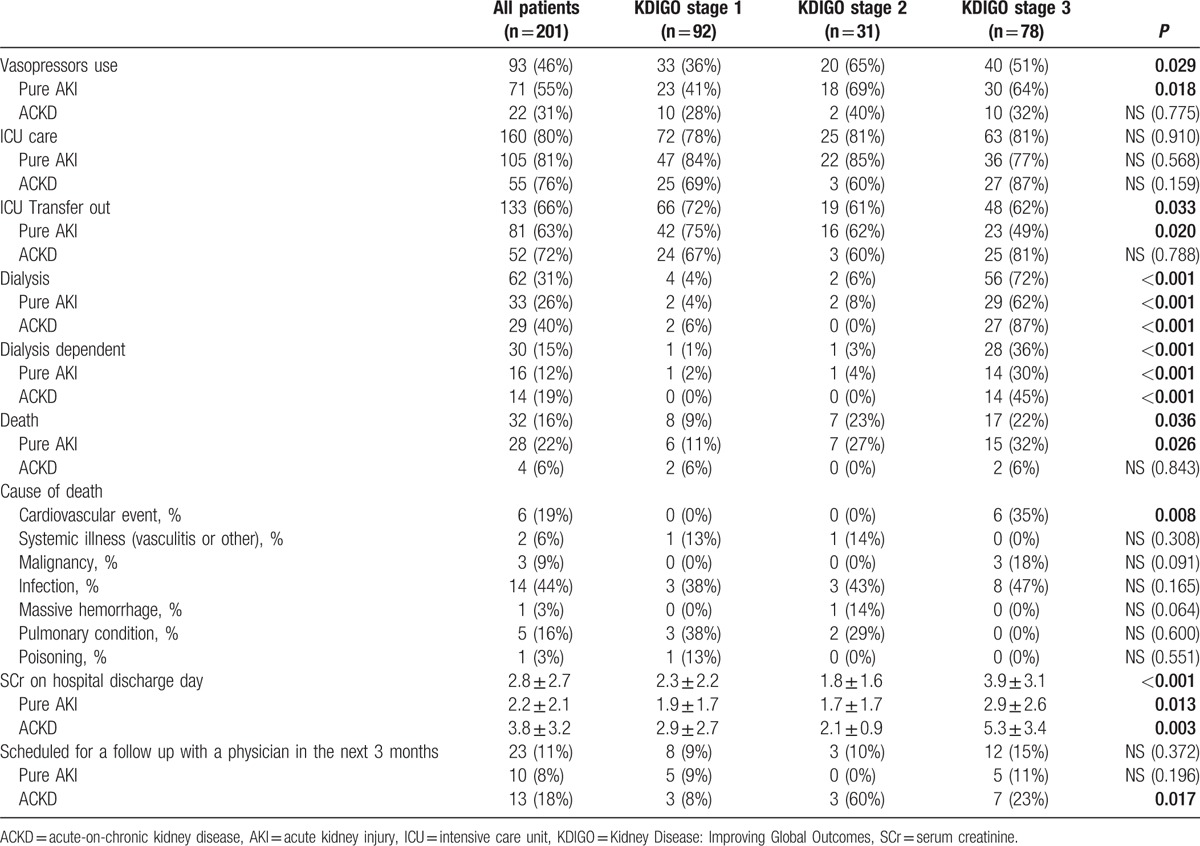
Management and outcomes according to AKI severity on admission.

**Figure 1:**
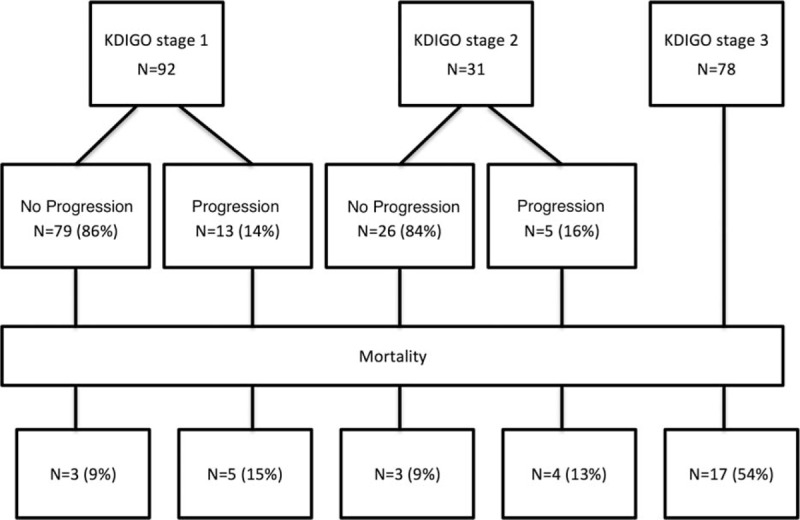
Prognosis of patients according to various AKI severity levels. Most patients with KDIGO stages 1 and 2 demonstrated stable or improved renal function. KDIGO stage 3 accounted for 54% of mortality. AKI = acute kidney injury, KDIGO = Kidney Disease: Improving Global Outcomes.

**Figure 2:**
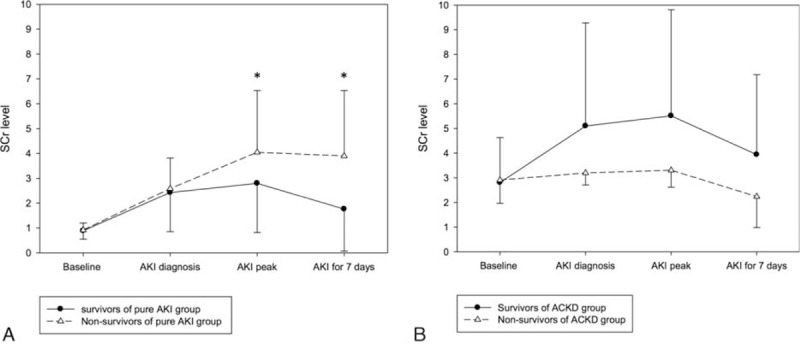
Changes in SCr levels within 7 days after AKI diagnosis in the (A) pure AKI and (B) ACKD groups. (A) In the pure AKI group, the SCr level did not differ between survivors and nonsurvivors at AKI onset. At subsequent time points, the SCr level increased significantly in nonsurvivors but not in survivors. (B) In the ACKD group, the SCr levels in nonsurvivors were persistently lower than those in survivors since AKI onset. ∗*P* < 0.05. ACKD = acute-on-chronic kidney disease, AKI = acute kidney injury, SCr = serum creatinine.

Figure 1 illustrates the prognosis of AKI patients in this study. KDIGO stage 3 accounted for 54% of mortality. As shown in Fig. 2A, the SCr levels significantly increased within 7 days after AKI diagnosis in nonsurvivors but not in survivors in the pure AKI group. By contrast, the SCr levels were lower in nonsurvivors than in survivors in the ACKD group during the same period, as shown in Fig. 2B.

## 4 Discussion

### 4.1 Main findings

We analyzed 201 hospitalized AKI patients in Taiwan to study the differences in clinical characteristics, management, and outcomes between AKI patients with and without preexisting CKD. Several critical risk factors for in-hospital mortality in AKI patients were identified. The main findings were as follows: 1st, overall in-hospital mortality was 15.9% (32/201). The in-hospital mortality rate of pure AKI patients was 21.7% (28/129), which was 3.82-fold higher than that of ACKD patients (5.6%, 4/72). Second, the preexisting CKD and AKI attributable to nephrotoxic agents were independent protective factors against in-hospital mortality. Oliguria occurrence at AKI diagnosis was an independent risk factor for in-hospital mortality. Third, the clinical courses of pure AKI and ACKD patients within 7 days after AKI diagnosis were significantly different. In pure AKI patients, relative changes in SCr levels (rather than absolute SCr levels at AKI diagnosis) were significantly associated with survival, whereas in ACKD patients, lower SCr levels during this period were associated with poor prognosis (Fig. 2, Table 2).

### 4.2 Comparison of clinical course and prognosis between AKI patients with and without preexisting CKD

In the literature, compared to patients without preexisting CKD, those with preexisting CKD have increased risk of developing AKI because of multiple comorbidities, impaired autoregulation, abnormal vasodilation response, and poor tolerability to side effects of medication.^[20,21]^ In this study, the ACKD patients had older age and more baseline risk factors for AKI. Chronic liver disease is a common cause of hepatic failure, and the occurrence of hepatic failure with AKI generally indicates a poor prognosis.^[22,23]^ However, for the high prevalence of diabetes and other chronic comorbidities, the prevalence of chronic liver disease seemed to be low in the ACKD patients. The clinical impact of chronic liver disease was more obvious in pure AKI group than in ACKD group (Table 1). Nevertheless, even excluding the patients with chronic liver disease or hepatic failure, the mortality rate of the pure AKI group was significantly higher than that of the ACKD group (The mortality rate of patients without chronic liver disease, pure AKI group vs ACKD group = 22.3% vs 5.6%, *P* = 0.003; The mortality rate of patients without hepatic failure, pure AKI group vs ACKD group = 18.0% vs 4.5%, *P* = 0.009). Interestingly, in spite of having poorer outcome, the pure AKI patients had lower rate of nephrologist consultation at AKI diagnosis and lower rate of scheduled medical follow-up within next 3 months after discharge.

Many studies have documented that even a minor fluctuation in the SCr level appears to be strongly associated with adverse outcomes,^[8,15,24]^ and studies have proposed the inclusion of small changes in SCr levels in the AKI definition in the Acute Kidney Injury Network and KDIGO criteria to enable earlier recognition of AKI episodes and early interventions. Notably, certain studies have reported that higher SCr levels are associated with a lower risk of mortality.^[25–30]^ Khosla et al^[19]^ and Cerda et al^[29]^ have described that preexisting CKD was associated with better survival in critically ill AKI patients. The strength of this multicenter study is that we prospectively investigated a wide variety of clinical settings for AKI. Our results reveal that the SCr level in survivors was significantly higher than that in nonsurvivors at the baseline, AKI diagnosis, peak of AKI, and before dialysis initiation. This trend was reversed at hospital discharge (SCr in survivors vs nonsurvivors = 2.6 ± 2.6 vs 3.7 ± 2.5, *P* = 0.049). When the overall patients were further stratified into pure AKI and ACKD groups, the paradoxical trend of higher SCr levels and better survival during admission was absent in the pure AKI group but persistent in the ACKD group. The aforementioned findings demonstrate the high impact of preexisting CKD on this paradoxical association of SCr and patient survival. In this investigation, the ACKD patients exhibited impaired fluid adjustment ability and were more susceptible to fluid overload, although they experienced less fluid gain than the pure AKI patients. Previous studies had reported the negative impact of fluid overload to patient survival,^[31]^ and our study had consistent results (Table 2). In addition, fluid overload might have blunted the rise in SCr in affected patients (correlation between SCr and estimated fluid gain on AKI confirmed day: *P* = 0.037). This may, at least partially, explain the strongly negative correlation of SCr and mortality in this study (Tables 2 and 3 and Fig. 2)

Perinel et al^[32]^ had reported that the duration of AKI could reflect its severity, this finding was consistent with our investigation. In this study, we further identified that the clinical courses of the pure AKI and ACKD groups were dissimilar. In overall patients and the subgroup of the pure AKI patients, the initial KDIGO staging and relative changes in SCr levels within 7 days after AKI diagnosis strongly influenced the survival chances (Tables 4 and 5, Figs. 1 and 2). This finding demonstrates the influence of the recovery or deterioration of renal function on mortality. Although in the ACKD patients, SCr levels in nonsurvivors were persistently lower than those in survivors since AKI diagnosis and the increased KDIGO staging was not significantly associated with increased risk of adverse outcomes in these patients (Table 5, Fig. 2). This finding signifies the higher impact of fluid overload and malnutrition in these patients.

### 4.3 Risk factors and protective factors of adverse outcomes among AKI patients

AKI is common in hospitalized patients, and its occurrence is associated with higher mortality and risks of CKD.^[4,33–37]^ In the present study, oliguria, preexisting CKD, and exposure to nephrotoxic agents were critical factors associated with increased or decreased in-hospital mortality among AKI patients.

Oliguria occurrence is commonly followed by fluid overload, electrolyte imbalance, and acid–base disturbance as well as impacts on multiple organ systems and immune function through the accumulation of fluid and metabolic by-products.^[38]^ These adverse events are clearly associated with a poor outcome. Nephrotoxic agent-related AKI has always been a specific cause of renal impairment. Stopping nephrotoxic agents, avoiding further exposure, and enhancing their excretion are crucial in the management of nephrotoxic agent-related AKI. These measures can prevent further renal deterioration and subsequent poor prognosis. Notably, our analysis results reveal that preexisting CKD was a protective factor against mortality in AKI patients. This finding is explained as follows reasons: 1st, although the molecular mechanisms of impairment or recovery of organ function are still controversial, inhibition of the epithelial–mesenchymal transition (EMT) was recently reported to reverse fibrotic processes in disease progression.^[39]^ Modulation of the immune system might play a role in the adaptive mechanism. There are intrinsic mechanisms of kidney when exposed to toxic or ischemic insults, which protect it against subsequent insults.^[40,41]^ Furthermore, inflammation-induced damage has been proposed to depend essentially on the intensity of the inflammatory response as well as on the intrinsic capacity of host organs to tolerate the effects of the inflammatory response. Therefore, reducing the tolerance capacity of vital organs might reduce tissue damage engendered by acute insults.^[42]^ Second, patients with preexisting CKD were more susceptible to oliguria, fluid overload, electrolyte or acid–base disturbances, and uremia, and they fulfilled the criteria of dialysis or severe AKI, although they were exposed to less severe insults.^[29]^ Third, a previous study reported that delayed nephrology consultation was associated with poor prognosis in the setting of acute renal failure, regardless of whether dialysis was required.^[43]^ Compared with pure AKI patients, ACKD patients were more frequently managed by nephrologists in our study; this might partially contribute to improved prognosis, although it was difficult to demonstrate the possible benefits from early recognition of or timely intervention for AKI by nephrologists in this study. Fourth, even normal stress may result in small changes in SCr levels in CKD patients. One shortcoming of the current AKI definition is that it does not distinguish the physiological fluctuation in the SCr level from the pathological deterioration of renal function in CKD patients, and this might overestimate the incidence of AKI and underestimate its mortality rate in these patients.

The recognition of beneficial and risk factors of mortality among AKI patients may provide improved predictions of outcomes along with objective information for risk stratification and clinical decision making.

### 4.4 Potential clinical implications

Because a considerable number of hospitalized patients develop AKI in a wide variety of clinical settings and because it might not be accompanied by obvious symptoms, its timely diagnosis is challenging for investigators and clinicians. KDIGO classification aimed to standardize the definition of AKI and facilitate advances in the clinical practice and research.^[44]^ However, the influence of urine criteria and preexisting CKD on KDIGO-AKI staging has yet to be extensively described in the literature.^[9,12,14,45–48]^ Our study highlighted the contribution of urine criteria and the impact of preexisting CKD for the current AKI definition. Furthermore, we also found that the clinical course and prognosis were different between patients with pure AKI and ACKD. Early recognition of and timely intervention for AKI might be beneficial to pure AKI patients, and monitoring the fluid status and preventing fluid overload might be even more valuable to ACKD patients. On the basis of our study results, we suggest that future studies include preexisting CKD for risk prediction, AKI definition, and treatment protocol establishment. This may lead to improved patient outcomes.

### 4.5 Study limitations

This study has several potential limitations, despite its encouraging results. First, we used creatinine- and urine-output-based criteria (the KDIGO criteria) for diagnosing AKI. Blood loss, hemodilution, and premorbid conditions may affect the diagnosis of AKI. Second, because of the multicenter design of this study, the decision-making process might differ between the medical centers; therefore, bias in choices of medical utilization might exist and confound risk factor analysis. Nevertheless, the identified risk factors are comparable to those in previous studies. Third, the predictive accuracy of logistic regression had its own limitations.

## 5 Conclusion

The overall in-hospital mortality rate of hospitalized AKI patients was 15.9% in this nationwide multicenter prospective survey in Taiwan. The prognosis and clinical course of ACKD patients were significantly superior to those of pure AKI patients. This study highlights the shortcomings of the current AKI definition in the KDIGO criteria, and this may contribute to the future development of AKI staging systems or treatment protocols. Considering preexisting CKD in the AKI staging system may improve prognosis prediction and provide objective information for clinical decision making and medical resource allocation.

## Acknowledgments

The authors thank the National Clinical Trial Centre of National Taiwan University Hospital and the National Science Council, Executive Yuan, ROC, Taiwan (grant number, NSC 100-2325-B-002-063) for the support. The authors also thank all participants of CAKs as well as the staff of the National Health Research Institute and the Harvard Statistics. (The details of the members of CAKs can be found in the URL:).

## Supplementary Material

Supplemental Digital Content
