# Supplementary material for: A nationwide survey of clinical characteristics, management, and outcomes of acute kidney injury (AKI) – patients with and without preexisting chronic kidney disease have different prognoses
Source: Medicine (Baltimore). 2016 Sep 30;95(39):e4987. doi: 10.1097/MD.0000000000004987 (PMC5265947; doi:10.1097/MD.0000000000004987)
Supplement: Supplemental Digital Content [file medi-95-e4987-s001.docx]

The members of CAKs are listed according to their affiliation names in alphabetical order:

[Cardinal Tien Hospital]: Kuo-Cheng Lu, MD. [Chi-Mei Medical Center Liouying]: Jian-Jhong Wang, MD. [Chi-Mei Medical Center Yongkang]: Wei-Chih Kan, MD. [China Medical University Hospital]: Chiu-Ching Huang, MD, Che-Yi Chou, MD, PhD., Ya-Fei Yang, MD. [Dalin Tzu-Chi Hospital]: Jen-Pi Tsai, MD. PhD. [Far Eastern Memorial Hospital]: Hung-Yuan Chen, MD. [Hualien Tzu Chi Hospital]: Bang-Gee Hsu, MD, PhD. [International-Harvard Statistical Consulting Company]: Fu-Chang Hu, PhD. [Kaohsiung Chang Gung Memorial Hospital]: Chien-Te Lee, MD, PhD., Jin-Bor Chen, MD., Chih-Hsiung Lee, MD, Wen-Chin Lee, MD, PhD., Lung-Chih Li, MD, PhD., Te-Chuan Chen, MD. [Kaohsiung Medical University

Chung-Ho Memorial Hospital]: Hung-Chun Chen, MD, PhD., Shang-Jyh Hwang, MD., Mei-Chuan Kuo, MD. [Kaohsiung Municipal Ta-Tung Hospital]: Hugo You-Hsien Lin, MD. [Keelung Chang Gung Memorial Hospital]: Chin-Chan Lee, MD., Chiao-Yin Sun, MD., Heng-Chih Pan, MD. [Linkou Chang Gung Memorial Hospital]: Yung-Chang Chen, MD., Ming-Yang Chang, MD, PhD., Chang-Chyi Jenq, MD., Chan-Yu Lin, MD, PhD., Chih-Hsiang Chang, MD., Tsung-Yu Tsai, MD. [Lin-Shin Hospital]: Cheng-Min Chen, MD. [Luodong Saint Mary’s Hospital]: Chih-Chung Shiao, MD. [Mackay Memorial Hospital]: Chih-Jen Wu, MD, PhD.,

Cheng-Jua Lin, MD., Pei-Chen Wu, MD. [Mackay Memorial Hospital Taitung Branch]: Feng-Chi Kuo, MD. [Min-Sheng General Hospital]: Chih-Jen Weng, MD. [National Health Research Institutes]: Li-Kwang Chen, PhD. [National Taiwan University Hospital]: Kwan-Dun Wu, MD, PhD., Tzong-Shinn Chu. MD, PhD., Shuei-Liong Lin, MD, PhD., Vin-Cent Wu, MD, PhD., Chun-Fu Lai, MD, PhD. [National Taiwan University Hospital Bei-Hu Branch]: Tai-Shuan Lai, MD, PhD. [National Taiwan University Hospital Hsin-Chu Branch]: Wei-Shun Yang, MD.

[National Taiwan University Hospital Yun-Lin Branch]: Yung-Ming Chen, MD., Tao-Min Huang, MD. [New Taipei City Hospital Sanchong Branch]: Wen-Ding Hsu, MD, MS. [Shin-Kong Wo Ho-Su Memorial Hospital]: Jyh-Gang Leu, MD, PhD., Jui-Ting Chang MD. [Sin-Ren Hospital]: Hung-Hsiang Liou, MD. [Taichung Veteran General Hospital]: Kuo-Hsiung Hsu, MD. Ming-Ju Wu, MD, PhD., Chun-Te Huang, MD. [Taichung Veteran General Hospital Chiayi Branch]: Zi-hong You, MD. [Taipei City Hospital Heping Branch]: Chao-Fu Chang, MD. [Taipei Medical University Hospital]: Tzen-Wen Chen, MD. PhD., Hsi-Hsien Chen, MD. PhD., Fan-Chi

Chang, MD. PhD., Yen-Chung Lin, MD., Mai-Szu Wu, MD., Chih-Chin Kao, MD. [Taipei Tzu Chi Hospital]: Szu-Chun Hung, MD., Ko-Lin Kuo, MD, PhD., Che-Hsiung Wu, MD. [Taipei Veterans General Hospital]: Der-Cherng Tarng, MD, PhD., Wu-Chang Yang, MD., Chih-Yu Yang, MD, PhD., Kuo-Hua Lee, MD. [Taoyuan General Hospital, Ministry of Health and Welfare]: Wei-Jie Wang, MD, PhD., Sheng-Wen Ko, MD., Jui-Hsiang Lin, MD.
